# Chemo- and regioselective [3 + 2]-cyclo­additions of thio­carbonyl ylides: crystal structures of *trans*-8-benzoyl-1,1,3,3-tetra­methyl-7-tri­fluoro­methyl-5-thia­spiro­[3.4]octan-2-one and *trans*-3-benzoyl-2,2-diphenyl-4-(tri­fluoro­meth­yl)tetra­hydro­thio­phene

**DOI:** 10.1107/S2056989018015335

**Published:** 2018-11-06

**Authors:** Anthony Linden, Grzegorz Mlostoń, Paulina Grzelak, Heinz Heimgartner

**Affiliations:** a Department of Chemistry, University of Zurich, Winterthurerstrasse 190, CH-8057 Zurich, Switzerland; b Department of Organic and Applied Chemistry, University of Łódź, Tamka 12, PL-91-403 Łódź, Poland

**Keywords:** crystal structure, tetra­hydro­thio­phene, [3 + 2]-cyclo­addition

## Abstract

The title compounds, C_19_H_21_F_3_O_2_S and C_24_H_19_F_3_OS, were prepared *via* chemo- and regioselective [3 + 2]-cyclo­additions of the respective thio­carbonyl ylides (thio­carbonyl *S*-methanides), generated *in situ*, with (*E*)-4,4,4-tri­fluoro-1-phenyl­but-2-en-1-one. The thio­phene ring in the crystal structure of each compound has an envelope conformation. The largest differences between the two mol­ecular structures is in the bond lengths about the quaternary C atom of the thio­phene ring; in the spiro­cyclic structure, the C—C bonds to the spiro C atom in the cyclo­butane ring are around 1.60 Å, although this is also observed in related structures. In the same structure, weak inter­molecular C—H⋯*X* (*X* = S, O) inter­actions link the mol­ecules into extended ribbons running parallel to the [001] direction. In the other structure, weak C—H⋯π inter­actions link the mol­ecules into sheets parallel to the (010) plane.

## Abstract 2

The title compounds were prepared *via* chemo- and regioselective [3 + 2]-cyclo­additions. The thio­phene ring in each crystal structure has an envelope conformation. The largest differences between the two mol­ecular structures is in the bond lengths about the quaternary C atom of the thio­phene ring. In the spiro­cyclic structure, the C—C bonds to the spiro C atom in the cyclo­butane ring are around 1.60 Å and weak inter­molecular C—H⋯*X* (*X* = S, O) inter­actions link the mol­ecules into extended ribbons. In the other structure, weak C—H⋯π inter­actions link the mol­ecules into sheets.

## Chemical context   

Tetra­hydro­thio­phenes constitute a group of five-membered non-aromatic sulfur heterocycles and one of the most prominent representatives is biotin (Mistry & Dakshinamurti, 1964 ▸). In a series of our publications, we demonstrated that the [3 + 2]-cyclo­addition of *in situ*-generated thio­carbonyl *S*-methanides with activated electron-deficient ethenes is the method of choice for the preparation of differently substituted tetra­hydro­thio­phenes (Huisgen *et al.*, 1984 ▸; Mlostoń & Heimgartner, 2000 ▸). Recently, alternative methods have been published in a series of reports demonstrating the ongoing inter­est in their synthesis (Zamberlan *et al.*, 2018 ▸). For example, Lewis acid-catalysed reactions of thio­carbonyl compounds with ‘donor–acceptor cyclo­propanes’ have been reported (Augustin *et al.*, 2017 ▸; Matsumoto *et al.*, 2018 ▸). In addition, radical cyclizations (Ram *et al.*, 2016 ▸) and ‘sulfur Michael/Henry reactions’ (Zhang *et al.*, 2018 ▸) were elaborated as new approaches to tetra­hydro­thio­phenes. Furthermore, analogous domino reactions, *i.e*. ‘sulfa-Michael/Aldol reactions’ (Duan *et al.*, 2017 ▸) and ‘double Michael reactions’ (Meninno *et al.*, 2017 ▸) as well as ‘Michael–Henry–Cascade–Rearrangement reactions’ (Wang *et al.*, 2018 ▸) as asymmetric syntheses of highly substituted mono- and spiro­cyclic tetra­hydro­thio­phene derivatives have been described.
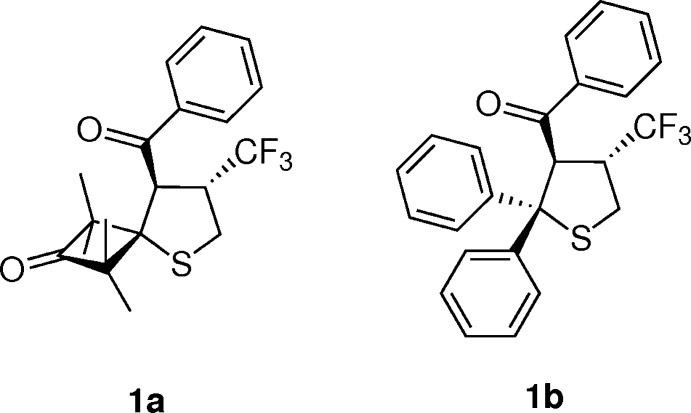


1,4-Disubstitued α,β-unsaturated ketones are known as reactive dipolarophiles, and in the case of aryl,trifluormethyl-substituted representatives, the [3 + 2]-cyclo­additions with electron-rich thio­carbonyl ylides occur chemoselectively either on the C=C or C=O bond, depending on the location of the CF_3_ group. In addition, the non-symmetrically substituted C=C bond can react with a thio­carbonyl *S*-methanide to give two different regioisomeric tetra­hydro­thio­phenes. We recently reported that the addition of the 1,3-dipole onto the C=C bond occurs only in the case of (*E*)-1-aryl-4,4,4-tri­fluoro­but-2-en-1-ones. On the other hand, the isomeric (*E*)-4-aryl-1,1,1-tri­fluoro­but-3-en-2-ones undergo cyclo­addition with the same thio­carbonyl *S*-methanide to afford 1,3-oxa­thiole derivatives exclusively (Mlostoń *et al.*, 2016 ▸). In that work, the [3 + 2]-cyclo­additions of (*E*)-4,4,4-tri­fluoro-1-phenyl­but-2-en-1-one with thio­benzo­phenone *S*-methanide as well as with 3-thioxo-2,2,4,4-tetra­methyl­cyclo­butan-3-one *S*-methanide led to the corresponding title tetra­hydro­thio­phene derivatives, *trans*-8-benzoyl-1,1,3,3-tetra­methyl-7-tri­fluoro­methyl-5-thia­spiro­[3.4]octan-2-one, **1a**, and *trans*-3-benzoyl-2,2-diphenyl-4-(tri­fluoro­meth­yl)tetra­hydro­thio­phene, **1b**, respectively, as crystalline products in high yields. Single crystals were grown from petroleum ether and used for single-crystal X-ray diffraction analyses, the results of which are reported here.

## Structural commentary   

**Figure 1:**
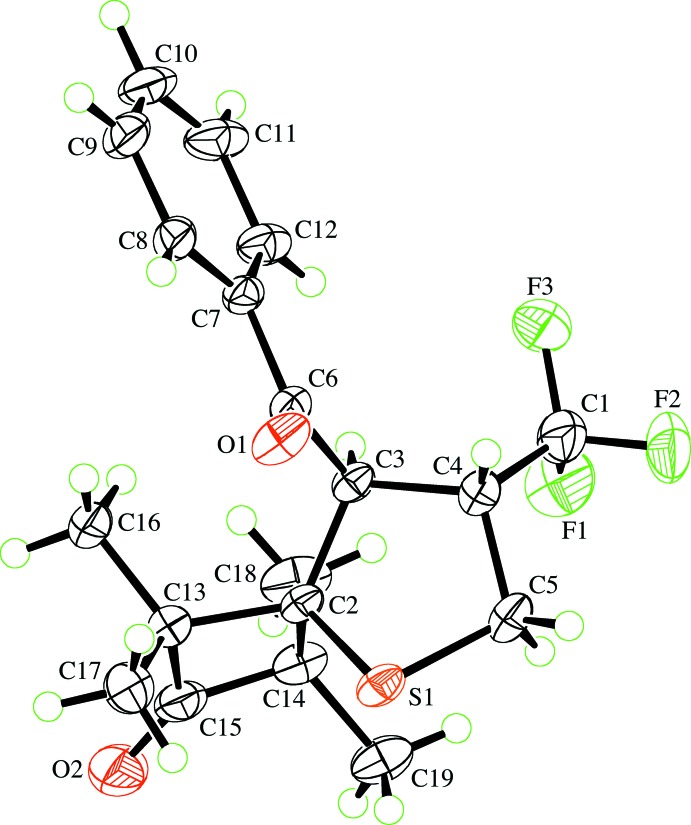
View of the mol­ecule of **1a** showing the atom-labelling scheme. Displacement ellipsoids are drawn at the 50% probability level. H atoms are represented by circles of arbitrary size.

Compounds **1a** and **1b** crystallized as racemates with the benzoyl and tri­fluoro­methyl substituents on the thio­phene ring in a *trans* configuration (Figs. 1 ▸ and 2 ▸). The thio­phene ring in each case has an envelope conformation with the sulfur atom as the envelope flap. For **1a**, the ring puckering parameters (Cremer & Pople, 1975 ▸) for the atom sequence S1,C2–C5 are *Q*(2) = 0.5164 (14) Å, φ(2) = 359.73 (18)° and atom S1 is 0.853 (1) Å from the mean plane through the other four ring atoms. The corresponding puckering parameters for **1b** are *Q*(2) = 0.5714 (16) Å, φ(2) = 349.86 (19)° with atom S1 being 0.921 (1) Å from the mean plane through the other four ring atoms. These parameters show that the thio­phene ring is slightly more distorted from an ideal envelope conformation in **1b** than in **1a**.

The most significant differences in the bond lengths within the two mol­ecules appears at the spiro C atom, C2 (Table 1 ▸). The C2—C13 and C2—C14 bonds involving the cyclo­butane ring in **1a**, at around 1.60 Å, are significantly longer than is usual for an alkyl C—C bond and 0.058 (3) and 0.072 (3) Å, respectively, longer than the corresponding bonds to the phenyl rings in **1b**. In concert, the S1—C2 and C2—C3 bonds are around 0.034 (2) Å shorter and the C3—C4 bond 0.018 (3) Å longer in **1a** than in **1b**; all other related bond lengths in the two mol­ecules are comparable. Despite these variations and the acute ‘bite angle’ of the cyclo­butane ring at C2 of the thio­phene ring [89.45 (12)° compared with 110.44 (14)° for the diphenyl-substituted **1b**], the intra-ring bond angles in the thio­phene rings of the two compounds are not very different. The above-mentioned differences in ring puckering presumably allow the bond-length variations not to impinge on the intra-ring angles. The Cambridge Structural Database (CSD, Version 5.39 with August 2018 updates; Groom *et al.*, 2016 ▸) contains one other example of a 2-cyclo­butane-substituted thio­phene ring (Seyfried *et al.*, 2006 ▸) and six examples of a 2,2-diphenyl-substituted thio­phene ring (Huisgen *et al.*, 1986 ▸; Seyfried *et al.*, 2006 ▸; Augustin *et al.*, 2017 ▸). These seven structures display exactly the same relative patterns of bond lengths as that described above.

The carbonyl group in **1b** is significantly twisted out of the plane of the benzoyl ring, with the O1—C6—C7—C8 torsion angle being −9.1 (2) and −29.5 (3)° in **1a** and **1b**, respectively. The O1—C6—C3—C4 torsion angles also differ by about 41°, so that the carbonyl group is more slanted with respect to the mean plane of the thio­phene ring in **1b** than in **1a**.

## Supra­molecular features   

**Figure 3:**
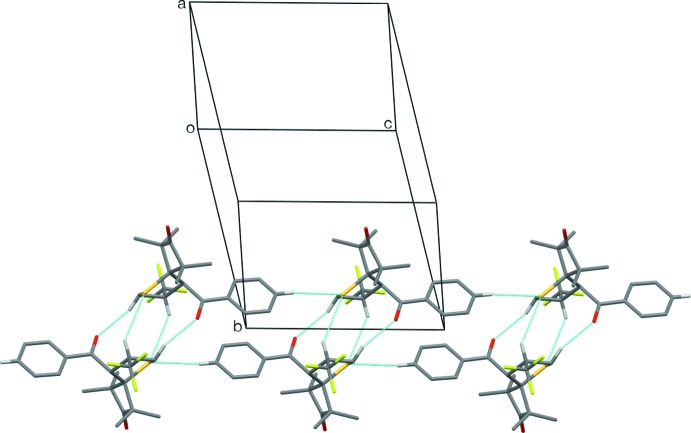
The ribbons formed by the weak inter­molecular C—H⋯*X* (*X* = S, O) inter­actions in **1a**. Most H atoms have been omitted for clarity.

In **1a**, there are three unique potentially significant weak supra­molecular contacts (Table 2 ▸). One of the methyl­ene H atoms at C5 inter­acts with the carbonyl O atom of a neighbouring mol­ecule related by a centre of inversion, while the methine H atom at the CF_3_-substituted C4 of this second mol­ecule inter­acts with the S atom of the first mol­ecule, thus forming centrosymmetric mol­ecular pairs with a total of four inter­actions between them. Graph-set motifs (Bernstein *et al.*, 1995 ▸) 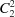
(8) (two different ones), 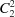
(9) and 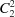
(12) can be discerned here. The third inter­action is a C—H⋯S inter­action between the *para*-H atom at C10 of the benzoyl ring and the S atom of a mol­ecule related by one unit-cell translation parallel to the [001] direction. This forms a chain of mol­ecules with a graph-set descriptor of *C*(9). The combination of these inter­actions leads to double-stranded chains of mol­ecules, or ribbons, running parallel to the [001] direction (Fig. 3 ▸). Within these ribbons, there is also a potential π–π inter­action between adjacent parallel benzoyl rings, where the centroid–centroid distance is 3.8740 (10) Å and the perpendicular distance between the ring planes is 3.4342 (7) Å, although the offset of the rings is rather large at 1.79 Å, so that the separation may be a fortuitous consequence of the alignment resulting from the other inter­actions.

**Figure 4:**
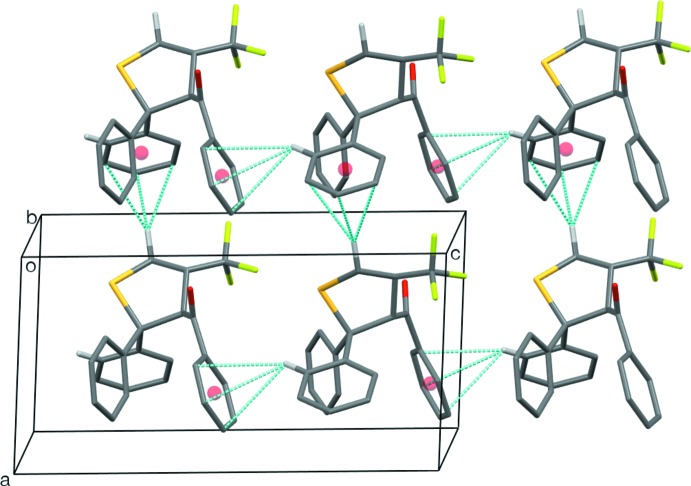
The sheets formed by the weak inter­molecular C—H⋯π inter­actions in **1b**. The relevant centroids are shown as red spheres. Most H atoms have been omitted for clarity.

In **1b**, the main supra­molecular features are two C—H⋯π inter­actions (Table 3 ▸): C24—H24 of one phenyl ring inter­acts with the benzoyl ring of a neighbouring mol­ecule related by a glide plane to give chains of mol­ecules parallel to the [001] direction, while one of the methyl­ene H atoms at C5 inter­acts with one of the phenyl rings in the mol­ecule related by one unit cell translation parallel to the [100] direction. Together, these inter­actions link the mol­ecules into sheets which lie parallel to the (010) plane (Fig. 4 ▸). Within these sheets, there is a potential inter­molecular C—H⋯F inter­action involving another phenyl ring H atom (C15—H15⋯F3^ii^), albeit with a rather sharp C—H⋯F angle of 121° [H15⋯F3^ii^ = 2.53 Å, C15⋯F3^ii^ = 3.132 (2) Å; symmetry code as in Table 4 ▸].

## Database survey   

The CSD contains crystal structure data with atomic coord­in­ates for 3225 monomeric organic compounds with the string *thio­phene* in the compound name, of which 70 are named as *tetra­hydro­thio­phenes* and 32 contain no substituents on the ring S atom. Recently published monocyclic crystal structures of tetra­hydro­thio­phenes include those of Duan *et al.* (2017 ▸), Ram *et al.* (2016 ▸), Zamberlan *et al.* (2018 ▸) and Zhang *et al.* (2018 ▸). Spiro­cyclic examples involving two cojoined five-membered rings have been reported by Meninno *et al.* (2017 ▸) and Wang *et al.* (2018 ▸).

## Synthesis and Crystallization   

**Figure 5:**
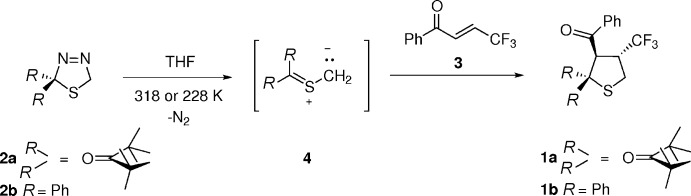
The reaction scheme leading to **1a** and **1b**.

The title compounds were prepared according to the reaction sequence presented in Fig. 5 ▸ and fully described with full spectroscopic data by Mlostoń *et al.* (2016 ▸). Thermal decomposition of 1,3,4-thia­diazo­lines **2a** and **2b** in THF solution in the presence of (*E*)-4,4,4-tri­fluoro-1-phenyl­but-2-en-1-one (**3**) leads to the tetra­hydro­thio­phenes **1a** and **1b**, respectively, as the product of the [3 + 2]-cyclo­addition of the inter­mediate thio­carbonyl *S*-methanides **4** with the activated C=C bond. Whereas the more stable **2a**, derived from 3-thioxo-2,2,4,4-tetra­methyl­cyclo­butanone, decomposes at 318 K, the less stable precursor **2b**, derived from thio­benzo­phenone, already extrudes N_2_ at 228 K. The ^1^H NMR analysis showed that only one product was formed in each case. After chromatographic purification, the isolated products were crystallized from petroleum ether by slow evaporation of the solvent.

## Refinement   

Crystal data, data collection and structure refinement details are summarized in Table 4 ▸. The methyl H atoms were constrained to an ideal geometry (C—H = 0.98 Å) with *U*_iso_(H) = 1.5*U*_eq_(C) while each group was allowed to rotate freely about its parent C—C bond. All other H atoms were placed in geometrically idealized positions and constrained to ride on their parent atoms with C—H distances in the range 0.95–1.00 Å and *U*_iso_(H) = 1.2*U*_eq_(C). For **1a**, one low angle reflection was omitted from the final cycles of refinement because its observed intensity was much lower than the calculated value.

## Supplementary Material

Crystal structure: contains datablock(s) 1a, 1b, global. DOI: 10.1107/S2056989018015335/vm2212sup1.cif

Structure factors: contains datablock(s) 1a. DOI: 10.1107/S2056989018015335/vm22121asup2.hkl

Supporting information file. DOI: 10.1107/S2056989018015335/vm22121asup4.cdx

Structure factors: contains datablock(s) 1b. DOI: 10.1107/S2056989018015335/vm22121bsup3.hkl

Supporting information file. DOI: 10.1107/S2056989018015335/vm22121bsup5.cdx

CCDC references: 1876143, 1876144

Additional supporting information:  crystallographic information; 3D view; checkCIF report

## Figures and Tables

**Figure 2:**
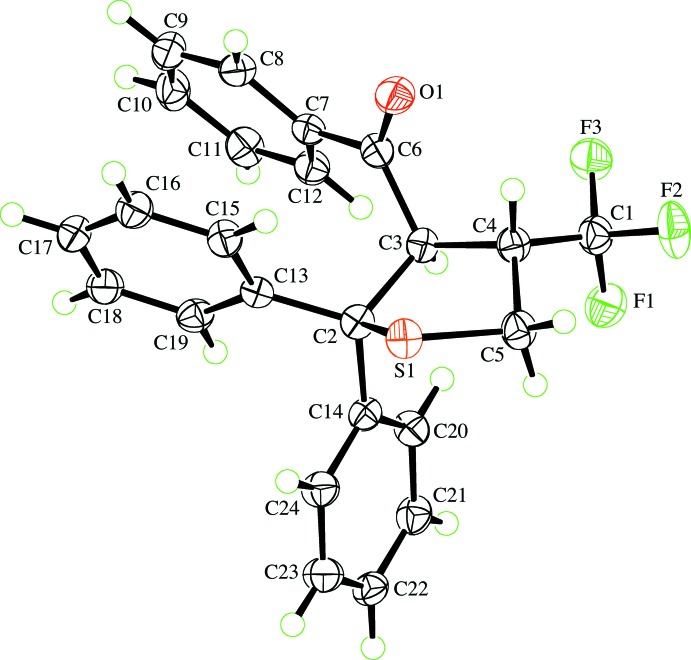
View of the mol­ecule of **1b** showing the atom-labelling scheme. Displacement ellipsoids are drawn at the 50% probability level. H atoms are represented by circles of arbitrary size.

**Table 1:** Comparison of selected geometric parameters (Å, °) for compounds **1a** and **1b**

Compound	**1a**	**1b**
S1—C2	1.8215 (14)	1.8567 (18)
S1—C5	1.7931 (17)	1.799 (2)
O1—C6	1.2187 (19)	1.215 (2)
C1—C4	1.493 (2)	1.503 (3)
C2—C3	1.556 (2)	1.590 (2)
C2—C13	1.592 (2)	1.534 (2)
C2—C14	1.609 (2)	1.537 (2)
C3—C4	1.562 (2)	1.544 (2)
C3—C6	1.529 (2)	1.532 (3)
C4—C5	1.537 (2)	1.533 (3)
C6—C7	1.495 (2)	1.495 (3)
		
C2—S1—C5	90.78 (7)	90.00 (8)
S1—C2—C3	104.75 (10)	103.16 (11)
S1—C2—C13	110.69 (10)	109.24 (12)
S1—C2—C14	112.84 (9)	107.10 (12)
C3—C2—C13	122.15 (12)	113.37 (14)
C3—C2—C14	116.78 (12)	113.04 (15)
C13—C2—C14	89.45 (12)	110.44 (14)
C2—C3—C4	108.37 (11)	108.88 (14)
C3—C4—C5	109.81 (13)	109.35 (15)
S1—C5—C4	105.22 (10)	102.77 (13)
		
C4—C3—C6—O1	−64.03 (18)	−23.0 (2)
O1—C6—C7—C8	−9.1 (2)	−29.5 (3)

**Table 2:** Hydrogen-bond geometry (Å, °) for **1a** [Chem scheme1]

*D*—H⋯*A*	*D*—H	H⋯*A*	*D*⋯*A*	*D*—H⋯*A*
C4—H4⋯S1^i^	1.00	2.89	3.7370 (16)	142
C5—H51⋯O1^i^	0.99	2.41	3.3417 (19)	156
C10—H10⋯S1^ii^	0.95	2.86	3.7970 (17)	171

Symmetry codes: (i) 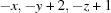; (ii) 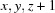.

**Table 3:** Weak C—H⋯π inter­actions (Å, °) found in **1b** *Cg*1 and *Cg*2 are the centroids of the C14,C20–C24 and C7–C12 rings, respectively.

	H⋯*Cg*	C⋯*Cg*	C—H⋯*Cg*
C5—H51⋯*Cg*1^i^	2.84	3.810 (2)	165
C24—H24—*Cg*2^ii^	2.86	3.625 (2)	139

Symmetry codes: (i) *x* − 1, *y*, *z*; (ii) *x*, −*y* + 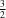, *z* − 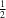.

**Table 4:** Experimental details

	**1a**	**1b**
Crystal data		
Chemical formula	C_19_H_21_F_3_O_2_S	C_24_H_19_F_3_OS
*M*_r_	370.42	412.45
Crystal system, space group	Monoclinic, *P*2_1_/*n*	Monoclinic, *P*2_1_/*c*
Temperature (K)	160	160
*a*, *b*, *c* (Å)	10.4851 (1), 15.4106 (2), 11.4557 (1)	7.4578 (1), 17.6162 (3), 14.5634 (2)
β (°)	103.8526 (7)	92.6805 (9)
*V* (Å^3^)	1797.19 (3)	1911.22 (5)
*Z*	4	4
Radiation type	Mo *K*α	Mo *K*α
μ (mm^−1^)	0.22	0.21
Crystal size (mm)	0.30 × 0.27 × 0.25	0.30 × 0.15 × 0.13

Data collection		
Diffractometer	Nonius KappaCCD area-detector	Nonius KappaCCD area-detector
Absorption correction	Multi-scan (Blessing, 1995 ▸)	Multi-scan (Blessing, 1995 ▸)
*T*_min_, *T*_max_	0.895, 0.949	0.904, 0.975
No. of measured, independent and observed [*I* > 2σ(*I*)] reflections	40775, 4120, 3322	43080, 4376, 3216
*R*_int_	0.053	0.081
(sin θ/λ)_max_ (Å^−1^)	0.650	0.650

Refinement		
*R*[*F* ^2^ > 2σ(*F* ^2^)], *wR*(*F* ^2^), *S*	0.043, 0.114, 1.06	0.045, 0.118, 1.07
No. of reflections	4119	4376
No. of parameters	231	263
H-atom treatment	H-atom parameters constrained	H-atom parameters constrained
Δρ_max_, Δρ_min_ (e Å^−3^)	0.32, −0.39	0.30, −0.37

Computer programs: *COLLECT* (Nonius, 2000 ▸), *DENZO-SMN* and *SCALEPACK* (Otwinowski & Minor, 1997 ▸), *SHELXS97* (Sheldrick, 2008 ▸), *ORTEPII* (Johnson, 1976 ▸), *SHELXL2018* (Sheldrick, 2015 ▸) and *PLATON* (Spek, 2015 ▸).

## supplementary crystallographic information

### trans-8-Benzoyl-1,1,3,3-tetramethyl-7-trifluoromethyl-5-thiaspiro[3.4]octan-2-one (1a) Crystal data

**Table:**

C_19_H_21_F_3_O_2_S	*D*_x_ = 1.369 Mg m^−^^3^
*M**_r_* = 370.42	Melting point: 387.3 K
Monoclinic, *P*2_1_/*n*	Mo *K*α radiation, λ = 0.71073 Å
*a* = 10.4851 (1) Å	Cell parameters from 41828 reflections
*b* = 15.4106 (2) Å	θ = 2.0–27.5°
*c* = 11.4557 (1) Å	µ = 0.22 mm^−^^1^
β = 103.8526 (7)°	*T* = 160 K
*V* = 1797.19 (3) Å^3^	Prism, colourless
*Z* = 4	0.30 × 0.27 × 0.25 mm
*F*(000) = 776	

### trans-8-Benzoyl-1,1,3,3-tetramethyl-7-trifluoromethyl-5-thiaspiro[3.4]octan-2-one (1a) Data collection

**Table:**

Nonius KappaCCD area-detector diffractometer	4120 independent reflections
Radiation source: Nonius FR590 sealed tube generator	3322 reflections with *I* > 2σ(*I*)
Horizontally mounted graphite crystal monochromator	*R*_int_ = 0.053
Detector resolution: 9 pixels mm^-1^	θ_max_ = 27.5°, θ_min_ = 2.3°
ω scans with κ offsets	*h* = −13→13
Absorption correction: multi-scan (Blessing, 1995)	*k* = −19→20
*T*_min_ = 0.895, *T*_max_ = 0.949	*l* = −14→14
40775 measured reflections	

### trans-8-Benzoyl-1,1,3,3-tetramethyl-7-trifluoromethyl-5-thiaspiro[3.4]octan-2-one (1a) Refinement

**Table:**

Refinement on *F*^2^	Hydrogen site location: inferred from neighbouring sites
Least-squares matrix: full	H-atom parameters constrained
*R*[*F*^2^ > 2σ(*F*^2^)] = 0.043	*w* = 1/[σ^2^(*F*_o_^2^) + (0.0563*P*)^2^ + 0.7139*P*] where *P* = (*F*_o_^2^ + 2*F*_c_^2^)/3
*wR*(*F*^2^) = 0.114	(Δ/σ)_max_ = 0.001
*S* = 1.06	Δρ_max_ = 0.32 e Å^−^^3^
4119 reflections	Δρ_min_ = −0.39 e Å^−^^3^
231 parameters	Extinction correction: SHELXL2018 (Sheldrick, 2015), Fc^*^=kFc[1+0.001xFc^2^λ^3^/sin(2θ)]^-1/4^
0 restraints	Extinction coefficient: 0.0172 (19)
Primary atom site location: structure-invariant direct methods	

### trans-8-Benzoyl-1,1,3,3-tetramethyl-7-trifluoromethyl-5-thiaspiro[3.4]octan-2-one (1a) Special details

**Table:**

Experimental. Data collection and full structure determination done by Prof. Anthony Linden: anthony.linden@chem.uzh.chSolvent used: petroleum ether Cooling Device: Oxford Cryosystems Cryostream 700 Crystal mount: on a glass fibre Client: Grzegorz Mloston Sample code: MG-1226 (HG1701)
Geometry. All esds (except the esd in the dihedral angle between two l.s. planes) are estimated using the full covariance matrix. The cell esds are taken into account individually in the estimation of esds in distances, angles and torsion angles; correlations between esds in cell parameters are only used when they are defined by crystal symmetry. An approximate (isotropic) treatment of cell esds is used for estimating esds involving l.s. planes.

### trans-8-Benzoyl-1,1,3,3-tetramethyl-7-trifluoromethyl-5-thiaspiro[3.4]octan-2-one (1a) Fractional atomic coordinates and isotropic or equivalent isotropic displacement parameters (Å^2^)

**Table:**

	*x*	*y*	*z*	*U*_iso_*/*U*_eq_	
S1	0.20161 (4)	0.95486 (3)	0.50667 (3)	0.02982 (13)	
F1	0.05271 (15)	0.72049 (7)	0.62746 (12)	0.0645 (4)	
F2	−0.13401 (13)	0.78097 (10)	0.55354 (12)	0.0703 (4)	
F3	−0.04964 (13)	0.78355 (9)	0.74481 (11)	0.0620 (4)	
O2	0.60425 (13)	0.88052 (12)	0.65763 (13)	0.0581 (4)	
O1	0.13585 (13)	1.03713 (7)	0.75063 (10)	0.0382 (3)	
C1	−0.0212 (2)	0.79034 (13)	0.63701 (17)	0.0456 (5)	
C2	0.28653 (15)	0.90617 (10)	0.64896 (12)	0.0250 (3)	
C3	0.17678 (14)	0.88890 (10)	0.71659 (12)	0.0244 (3)	
H3	0.199188	0.835746	0.767439	0.029*	
C4	0.04466 (15)	0.87394 (10)	0.62132 (13)	0.0298 (3)	
H4	−0.016358	0.921985	0.630370	0.036*	
C5	0.06769 (17)	0.88032 (11)	0.49408 (13)	0.0330 (4)	
H51	−0.011859	0.902266	0.436656	0.040*	
H52	0.090577	0.822850	0.466259	0.040*	
C6	0.15834 (14)	0.96546 (10)	0.79587 (13)	0.0258 (3)	
C7	0.16542 (14)	0.95313 (10)	0.92674 (13)	0.0268 (3)	
C8	0.16453 (15)	1.02830 (12)	0.99516 (15)	0.0340 (4)	
H8	0.158220	1.083677	0.957765	0.041*	
C9	0.17284 (17)	1.02194 (15)	1.11734 (16)	0.0453 (5)	
H9	0.173785	1.073012	1.163988	0.054*	
C10	0.17976 (17)	0.94132 (16)	1.17150 (15)	0.0492 (5)	
H10	0.185190	0.937279	1.255300	0.059*	
C11	0.17884 (18)	0.86679 (15)	1.10481 (15)	0.0450 (5)	
H11	0.182779	0.811594	1.142426	0.054*	
C12	0.17211 (16)	0.87256 (12)	0.98213 (14)	0.0344 (4)	
H12	0.172109	0.821221	0.936230	0.041*	
C13	0.41591 (16)	0.95942 (11)	0.70821 (14)	0.0325 (4)	
C14	0.37631 (16)	0.82468 (11)	0.63197 (14)	0.0326 (4)	
C15	0.49199 (17)	0.88639 (13)	0.66570 (14)	0.0378 (4)	
C16	0.45250 (17)	0.96613 (14)	0.84639 (15)	0.0414 (4)	
H161	0.402906	1.013595	0.871564	0.062*	
H162	0.546810	0.977600	0.874510	0.062*	
H163	0.431050	0.911455	0.881007	0.062*	
C17	0.4327 (2)	1.04824 (13)	0.65521 (17)	0.0457 (5)	
H171	0.420606	1.043024	0.567987	0.069*	
H172	0.521016	1.070364	0.690990	0.069*	
H173	0.367175	1.088436	0.672592	0.069*	
C18	0.3887 (2)	0.75333 (12)	0.72822 (17)	0.0448 (5)	
H181	0.403045	0.780143	0.807879	0.067*	
H182	0.463169	0.715555	0.725600	0.067*	
H183	0.307813	0.718887	0.712424	0.067*	
C19	0.3591 (2)	0.78204 (12)	0.50888 (16)	0.0420 (4)	
H191	0.438204	0.748806	0.506841	0.063*	
H192	0.344879	0.826937	0.446508	0.063*	
H193	0.283109	0.743019	0.494278	0.063*	

### trans-8-Benzoyl-1,1,3,3-tetramethyl-7-trifluoromethyl-5-thiaspiro[3.4]octan-2-one (1a) Atomic displacement parameters (Å^2^)

**Table:**

	*U*^11^	*U*^22^	*U*^33^	*U*^12^	*U*^13^	*U*^23^
S1	0.0407 (2)	0.0293 (2)	0.01836 (19)	0.00149 (16)	0.00501 (15)	0.00383 (14)
F1	0.0989 (10)	0.0296 (6)	0.0672 (8)	−0.0136 (6)	0.0241 (7)	−0.0031 (5)
F2	0.0639 (8)	0.0832 (10)	0.0567 (8)	−0.0422 (7)	0.0005 (6)	−0.0081 (7)
F3	0.0719 (8)	0.0713 (9)	0.0476 (7)	−0.0330 (7)	0.0235 (6)	0.0000 (6)
O2	0.0366 (7)	0.0939 (12)	0.0461 (8)	0.0053 (7)	0.0149 (6)	−0.0121 (8)
O1	0.0596 (8)	0.0268 (6)	0.0266 (6)	0.0075 (5)	0.0073 (5)	0.0008 (4)
C1	0.0551 (11)	0.0441 (11)	0.0375 (9)	−0.0184 (9)	0.0108 (8)	−0.0058 (8)
C2	0.0313 (8)	0.0263 (7)	0.0170 (6)	0.0009 (6)	0.0050 (5)	0.0010 (5)
C3	0.0309 (7)	0.0230 (7)	0.0186 (6)	0.0017 (6)	0.0041 (5)	0.0013 (5)
C4	0.0319 (8)	0.0308 (8)	0.0250 (7)	−0.0016 (6)	0.0031 (6)	−0.0023 (6)
C5	0.0395 (9)	0.0340 (9)	0.0216 (7)	−0.0005 (7)	−0.0003 (6)	−0.0022 (6)
C6	0.0268 (7)	0.0282 (8)	0.0212 (7)	0.0016 (6)	0.0038 (5)	−0.0009 (6)
C7	0.0214 (7)	0.0384 (9)	0.0199 (7)	0.0036 (6)	0.0037 (5)	−0.0013 (6)
C8	0.0271 (8)	0.0463 (10)	0.0284 (8)	−0.0008 (7)	0.0063 (6)	−0.0106 (7)
C9	0.0316 (9)	0.0751 (14)	0.0290 (9)	0.0006 (9)	0.0067 (7)	−0.0190 (9)
C10	0.0338 (9)	0.0956 (17)	0.0191 (7)	0.0113 (10)	0.0083 (6)	−0.0009 (9)
C11	0.0405 (10)	0.0701 (13)	0.0267 (8)	0.0167 (9)	0.0124 (7)	0.0158 (8)
C12	0.0340 (8)	0.0447 (10)	0.0254 (8)	0.0099 (7)	0.0091 (6)	0.0063 (7)
C13	0.0313 (8)	0.0420 (10)	0.0244 (7)	−0.0051 (7)	0.0074 (6)	−0.0031 (6)
C14	0.0401 (9)	0.0343 (9)	0.0245 (7)	0.0102 (7)	0.0101 (6)	0.0021 (6)
C15	0.0357 (9)	0.0567 (11)	0.0217 (7)	0.0056 (8)	0.0081 (6)	0.0007 (7)
C16	0.0309 (8)	0.0657 (13)	0.0260 (8)	−0.0057 (8)	0.0040 (6)	−0.0093 (8)
C17	0.0521 (11)	0.0458 (11)	0.0413 (10)	−0.0192 (9)	0.0151 (8)	−0.0049 (8)
C18	0.0599 (12)	0.0410 (10)	0.0369 (9)	0.0233 (9)	0.0183 (8)	0.0120 (8)
C19	0.0563 (11)	0.0392 (10)	0.0328 (9)	0.0122 (8)	0.0154 (8)	−0.0046 (7)

### trans-8-Benzoyl-1,1,3,3-tetramethyl-7-trifluoromethyl-5-thiaspiro[3.4]octan-2-one (1a) Geometric parameters (Å, º)

**Table:**

S1—C2	1.8215 (14)	C9—H9	0.9500
S1—C5	1.7931 (17)	C10—C11	1.378 (3)
F1—C1	1.346 (3)	C10—H10	0.9500
F2—C1	1.339 (2)	C11—C12	1.393 (2)
F3—C1	1.342 (2)	C11—H11	0.9500
O2—C15	1.206 (2)	C12—H12	0.9500
O1—C6	1.2187 (19)	C13—C17	1.525 (2)
C1—C4	1.493 (2)	C13—C15	1.525 (2)
C2—C3	1.556 (2)	C13—C16	1.540 (2)
C2—C13	1.592 (2)	C14—C15	1.517 (3)
C2—C14	1.609 (2)	C14—C19	1.527 (2)
C3—C4	1.562 (2)	C14—C18	1.541 (2)
C3—C6	1.529 (2)	C16—H161	0.9800
C3—H3	1.0000	C16—H162	0.9800
C4—C5	1.537 (2)	C16—H163	0.9800
C4—H4	1.0000	C17—H171	0.9800
C5—H51	0.9900	C17—H172	0.9800
C5—H52	0.9900	C17—H173	0.9800
C6—C7	1.495 (2)	C18—H181	0.9800
C7—C12	1.388 (2)	C18—H182	0.9800
C7—C8	1.400 (2)	C18—H183	0.9800
C8—C9	1.385 (2)	C19—H191	0.9800
C8—H8	0.9500	C19—H192	0.9800
C9—C10	1.383 (3)	C19—H193	0.9800
			
C2—S1—C5	90.78 (7)	C10—C11—C12	119.87 (19)
F2—C1—F3	107.18 (16)	C10—C11—H11	120.1
F2—C1—F1	106.45 (16)	C12—C11—H11	120.1
F3—C1—F1	105.77 (16)	C7—C12—C11	120.20 (17)
F2—C1—C4	111.22 (17)	C7—C12—H12	119.9
F3—C1—C4	112.90 (15)	C11—C12—H12	119.9
F1—C1—C4	112.88 (16)	C17—C13—C15	114.60 (14)
S1—C2—C3	104.75 (10)	C17—C13—C16	108.96 (15)
S1—C2—C13	110.69 (10)	C15—C13—C16	110.92 (14)
S1—C2—C14	112.84 (9)	C17—C13—C2	117.44 (14)
C3—C2—C13	122.15 (12)	C15—C13—C2	86.61 (12)
C3—C2—C14	116.78 (12)	C16—C13—C2	116.74 (13)
C13—C2—C14	89.45 (12)	C15—C14—C19	114.56 (14)
C6—C3—C2	112.18 (12)	C15—C14—C18	109.87 (15)
C6—C3—C4	108.16 (12)	C19—C14—C18	108.97 (15)
C2—C3—C4	108.37 (11)	C15—C14—C2	86.28 (12)
C6—C3—H3	109.4	C19—C14—C2	120.29 (13)
C2—C3—H3	109.4	C18—C14—C2	114.97 (13)
C4—C3—H3	109.4	O2—C15—C14	132.00 (18)
C1—C4—C5	110.59 (13)	O2—C15—C13	132.42 (18)
C1—C4—C3	113.68 (14)	C14—C15—C13	95.54 (13)
C3—C4—C5	109.81 (13)	C13—C16—H161	109.5
C1—C4—H4	107.5	C13—C16—H162	109.5
C5—C4—H4	107.5	H161—C16—H162	109.5
C3—C4—H4	107.5	C13—C16—H163	109.5
S1—C5—C4	105.22 (10)	H161—C16—H163	109.5
C4—C5—H51	110.7	H162—C16—H163	109.5
S1—C5—H51	110.7	C13—C17—H171	109.5
C4—C5—H52	110.7	C13—C17—H172	109.5
S1—C5—H52	110.7	H171—C17—H172	109.5
H51—C5—H52	108.8	C13—C17—H173	109.5
O1—C6—C7	119.97 (13)	H171—C17—H173	109.5
O1—C6—C3	119.06 (13)	H172—C17—H173	109.5
C7—C6—C3	120.96 (13)	C14—C18—H181	109.5
C12—C7—C8	119.37 (14)	C14—C18—H182	109.5
C12—C7—C6	123.84 (14)	H181—C18—H182	109.5
C8—C7—C6	116.79 (14)	C14—C18—H183	109.5
C9—C8—C7	120.00 (18)	H181—C18—H183	109.5
C9—C8—H8	120.0	H182—C18—H183	109.5
C7—C8—H8	120.0	C14—C19—H191	109.5
C10—C9—C8	120.06 (18)	C14—C19—H192	109.5
C10—C9—H9	120.0	H191—C19—H192	109.5
C8—C9—H9	120.0	C14—C19—H193	109.5
C11—C10—C9	120.49 (16)	H191—C19—H193	109.5
C11—C10—H10	119.8	H192—C19—H193	109.5
C9—C10—H10	119.8		
			
C5—S1—C2—C3	−40.88 (11)	C9—C10—C11—C12	0.6 (3)
C5—S1—C2—C13	−174.32 (11)	C8—C7—C12—C11	−0.5 (2)
C5—S1—C2—C14	87.17 (12)	C6—C7—C12—C11	179.82 (15)
C13—C2—C3—C6	35.81 (18)	C10—C11—C12—C7	−0.4 (3)
C14—C2—C3—C6	143.53 (13)	C3—C2—C13—C17	−111.55 (16)
S1—C2—C3—C6	−90.84 (12)	C14—C2—C13—C17	126.71 (15)
C13—C2—C3—C4	155.15 (13)	S1—C2—C13—C17	12.42 (18)
C14—C2—C3—C4	−97.12 (14)	C3—C2—C13—C15	132.39 (14)
S1—C2—C3—C4	28.50 (13)	C14—C2—C13—C15	10.65 (11)
F2—C1—C4—C5	−56.2 (2)	S1—C2—C13—C15	−103.63 (11)
F3—C1—C4—C5	−176.76 (15)	C3—C2—C13—C16	20.6 (2)
F1—C1—C4—C5	63.34 (19)	C14—C2—C13—C16	−101.10 (15)
F2—C1—C4—C3	179.72 (15)	S1—C2—C13—C16	144.61 (13)
F3—C1—C4—C3	59.2 (2)	C3—C2—C14—C15	−136.96 (12)
F1—C1—C4—C3	−60.71 (19)	C13—C2—C14—C15	−10.72 (11)
C6—C3—C4—C1	−113.10 (15)	S1—C2—C14—C15	101.58 (11)
C2—C3—C4—C1	125.06 (15)	C3—C2—C14—C19	106.66 (17)
C6—C3—C4—C5	122.43 (14)	C13—C2—C14—C19	−127.10 (16)
C2—C3—C4—C5	0.59 (17)	S1—C2—C14—C19	−14.8 (2)
C1—C4—C5—S1	−156.24 (13)	C3—C2—C14—C18	−26.7 (2)
C3—C4—C5—S1	−30.00 (15)	C13—C2—C14—C18	99.49 (16)
C2—S1—C5—C4	41.32 (11)	S1—C2—C14—C18	−148.21 (14)
C2—C3—C6—O1	55.44 (18)	C19—C14—C15—O2	−44.8 (3)
C4—C3—C6—O1	−64.03 (18)	C18—C14—C15—O2	78.2 (2)
C2—C3—C6—C7	−125.53 (14)	C2—C14—C15—O2	−166.6 (2)
C4—C3—C6—C7	115.00 (14)	C19—C14—C15—C13	132.98 (14)
O1—C6—C7—C12	170.63 (16)	C18—C14—C15—C13	−103.99 (14)
C3—C6—C7—C12	−8.4 (2)	C2—C14—C15—C13	11.24 (12)
O1—C6—C7—C8	−9.1 (2)	C17—C13—C15—O2	47.7 (3)
C3—C6—C7—C8	171.89 (14)	C16—C13—C15—O2	−76.2 (2)
C12—C7—C8—C9	1.2 (2)	C2—C13—C15—O2	166.4 (2)
C6—C7—C8—C9	−179.03 (14)	C17—C13—C15—C14	−130.09 (15)
C7—C8—C9—C10	−1.1 (3)	C16—C13—C15—C14	106.02 (14)
C8—C9—C10—C11	0.2 (3)	C2—C13—C15—C14	−11.36 (12)

### trans-8-Benzoyl-1,1,3,3-tetramethyl-7-trifluoromethyl-5-thiaspiro[3.4]octan-2-one (1a) Hydrogen-bond geometry (Å, º)

**Table:**

*D*—H···*A*	*D*—H	H···*A*	*D*···*A*	*D*—H···*A*
C4—H4···S1^i^	1.00	2.89	3.7370 (16)	142
C5—H51···O1^i^	0.99	2.41	3.3417 (19)	156
C10—H10···S1^ii^	0.95	2.86	3.7970 (17)	171

Symmetry codes: (i) −*x*, −*y*+2, −*z*+1; (ii) *x*, *y*, *z*+1.

### trans-3-Benzoyl-2,2-diphenyl-4-(trifluoromethyl)tetrahydrothiophene (1b) Crystal data

**Table:**

C_24_H_19_F_3_OS	*D*_x_ = 1.433 Mg m^−^^3^
*M**_r_* = 412.45	Melting point: 401.4 K
Monoclinic, *P*2_1_/*c*	Mo *K*α radiation, λ = 0.71073 Å
*a* = 7.4578 (1) Å	Cell parameters from 36099 reflections
*b* = 17.6162 (3) Å	θ = 2.0–27.5°
*c* = 14.5634 (2) Å	µ = 0.21 mm^−^^1^
β = 92.6805 (9)°	*T* = 160 K
*V* = 1911.22 (5) Å^3^	Prism, colourless
*Z* = 4	0.30 × 0.15 × 0.13 mm
*F*(000) = 856	

### trans-3-Benzoyl-2,2-diphenyl-4-(trifluoromethyl)tetrahydrothiophene (1b) Data collection

**Table:**

Nonius KappaCCD area-detector diffractometer	4376 independent reflections
Radiation source: Nonius FR590 sealed tube generator	3216 reflections with *I* > 2σ(*I*)
Horizontally mounted graphite crystal monochromator	*R*_int_ = 0.081
Detector resolution: 9 pixels mm^-1^	θ_max_ = 27.5°, θ_min_ = 2.3°
ω scans with κ offsets	*h* = −9→9
Absorption correction: multi-scan (Blessing, 1995)	*k* = −22→22
*T*_min_ = 0.904, *T*_max_ = 0.975	*l* = −18→18
43080 measured reflections	

### trans-3-Benzoyl-2,2-diphenyl-4-(trifluoromethyl)tetrahydrothiophene (1b) Refinement

**Table:**

Refinement on *F*^2^	Hydrogen site location: inferred from neighbouring sites
Least-squares matrix: full	H-atom parameters constrained
*R*[*F*^2^ > 2σ(*F*^2^)] = 0.045	*w* = 1/[σ^2^(*F*_o_^2^) + (0.053*P*)^2^ + 0.850*P*] where *P* = (*F*_o_^2^ + 2*F*_c_^2^)/3
*wR*(*F*^2^) = 0.118	(Δ/σ)_max_ = 0.001
*S* = 1.07	Δρ_max_ = 0.30 e Å^−^^3^
4376 reflections	Δρ_min_ = −0.37 e Å^−^^3^
263 parameters	Extinction correction: SHELXL2018 (Sheldrick, 2015), Fc^*^=kFc[1+0.001xFc^2^λ^3^/sin(2θ)]^-1/4^
0 restraints	Extinction coefficient: 0.0046 (10)
Primary atom site location: structure-invariant direct methods	

### trans-3-Benzoyl-2,2-diphenyl-4-(trifluoromethyl)tetrahydrothiophene (1b) Special details

**Table:**

Experimental. Data collection and full structure determination done by Prof. Anthony Linden: anthony.linden@chem.uzh.chSolvent used: petroleum ether Cooling Device: Oxford Cryosystems Cryostream 700 Crystal mount: on a glass fibre Client: Grzegorz Mloston Sample code: MG-1225 (HG1704)
Geometry. All esds (except the esd in the dihedral angle between two l.s. planes) are estimated using the full covariance matrix. The cell esds are taken into account individually in the estimation of esds in distances, angles and torsion angles; correlations between esds in cell parameters are only used when they are defined by crystal symmetry. An approximate (isotropic) treatment of cell esds is used for estimating esds involving l.s. planes.

### trans-3-Benzoyl-2,2-diphenyl-4-(trifluoromethyl)tetrahydrothiophene (1b) Fractional atomic coordinates and isotropic or equivalent isotropic displacement parameters (Å^2^)

**Table:**

	*x*	*y*	*z*	*U*_iso_*/*U*_eq_	
S1	0.28849 (6)	0.65984 (3)	0.67818 (3)	0.02909 (15)	
F1	0.31015 (18)	0.55032 (7)	0.95378 (9)	0.0437 (3)	
F2	0.03380 (16)	0.58579 (8)	0.94518 (9)	0.0483 (4)	
F3	0.23272 (16)	0.65536 (7)	1.01552 (8)	0.0389 (3)	
O1	0.29001 (18)	0.80937 (8)	0.86995 (11)	0.0357 (3)	
C1	0.2026 (3)	0.61134 (12)	0.94108 (14)	0.0313 (4)	
C2	0.4966 (2)	0.67383 (10)	0.75124 (12)	0.0240 (4)	
C3	0.4232 (2)	0.68733 (10)	0.85069 (12)	0.0237 (4)	
H3	0.502705	0.659132	0.896173	0.028*	
C4	0.2324 (2)	0.65367 (11)	0.85349 (13)	0.0261 (4)	
H4	0.145855	0.697070	0.850258	0.031*	
C5	0.1943 (3)	0.60455 (11)	0.76790 (13)	0.0298 (4)	
H51	0.063735	0.596885	0.756069	0.036*	
H52	0.253808	0.554471	0.774256	0.036*	
C6	0.4260 (2)	0.77143 (10)	0.87760 (13)	0.0261 (4)	
C7	0.6005 (2)	0.80580 (10)	0.91069 (12)	0.0256 (4)	
C8	0.6280 (3)	0.88269 (11)	0.89188 (13)	0.0304 (4)	
H8	0.534813	0.911678	0.862177	0.036*	
C9	0.7914 (3)	0.91680 (12)	0.91654 (14)	0.0349 (5)	
H9	0.811317	0.968572	0.901888	0.042*	
C10	0.9249 (3)	0.87515 (12)	0.96249 (14)	0.0351 (5)	
H10	1.036380	0.898546	0.979438	0.042*	
C11	0.8971 (3)	0.79961 (12)	0.98390 (14)	0.0327 (4)	
H11	0.988277	0.771614	1.016652	0.039*	
C12	0.7359 (2)	0.76487 (11)	0.95745 (13)	0.0284 (4)	
H12	0.717806	0.712819	0.971322	0.034*	
C13	0.6006 (2)	0.74187 (10)	0.71509 (12)	0.0236 (4)	
C14	0.6081 (2)	0.60080 (10)	0.74477 (13)	0.0245 (4)	
C15	0.5139 (3)	0.80438 (11)	0.67449 (13)	0.0288 (4)	
H15	0.386722	0.804491	0.666591	0.035*	
C16	0.6108 (3)	0.86634 (11)	0.64552 (13)	0.0311 (4)	
H16	0.549354	0.908407	0.617999	0.037*	
C17	0.7956 (3)	0.86759 (11)	0.65621 (14)	0.0319 (4)	
H17	0.861442	0.909989	0.635663	0.038*	
C18	0.8842 (3)	0.80624 (11)	0.69729 (14)	0.0313 (4)	
H18	1.011281	0.806856	0.705772	0.038*	
C19	0.7879 (2)	0.74429 (11)	0.72587 (13)	0.0276 (4)	
H19	0.850178	0.702451	0.753418	0.033*	
C20	0.6661 (2)	0.55616 (11)	0.81886 (13)	0.0286 (4)	
H20	0.635747	0.570169	0.879184	0.034*	
C21	0.7683 (2)	0.49105 (11)	0.80606 (14)	0.0305 (4)	
H21	0.806329	0.461316	0.857799	0.037*	
C22	0.8150 (2)	0.46917 (11)	0.71956 (14)	0.0294 (4)	
H22	0.883792	0.424547	0.711065	0.035*	
C23	0.7591 (3)	0.51386 (11)	0.64507 (14)	0.0334 (5)	
H23	0.790809	0.499834	0.584969	0.040*	
C24	0.6579 (3)	0.57844 (11)	0.65723 (13)	0.0296 (4)	
H24	0.621489	0.608249	0.605295	0.036*	

### trans-3-Benzoyl-2,2-diphenyl-4-(trifluoromethyl)tetrahydrothiophene (1b) Atomic displacement parameters (Å^2^)

**Table:**

	*U*^11^	*U*^22^	*U*^33^	*U*^12^	*U*^13^	*U*^23^
S1	0.0258 (2)	0.0342 (3)	0.0268 (3)	−0.00113 (19)	−0.00414 (18)	0.00193 (19)
F1	0.0556 (8)	0.0331 (7)	0.0430 (7)	0.0025 (6)	0.0071 (6)	0.0098 (5)
F2	0.0357 (7)	0.0685 (9)	0.0408 (7)	−0.0228 (6)	0.0049 (5)	0.0058 (6)
F3	0.0442 (7)	0.0441 (7)	0.0283 (6)	−0.0049 (5)	0.0006 (5)	−0.0035 (5)
O1	0.0272 (7)	0.0284 (7)	0.0516 (9)	0.0052 (6)	0.0038 (6)	0.0013 (6)
C1	0.0280 (10)	0.0341 (11)	0.0318 (11)	−0.0058 (8)	0.0026 (8)	−0.0014 (8)
C2	0.0227 (9)	0.0257 (9)	0.0233 (9)	0.0004 (7)	−0.0027 (7)	0.0010 (7)
C3	0.0215 (9)	0.0245 (9)	0.0248 (9)	−0.0005 (7)	−0.0009 (7)	0.0021 (7)
C4	0.0232 (9)	0.0285 (10)	0.0264 (10)	−0.0015 (7)	−0.0004 (7)	0.0001 (8)
C5	0.0251 (9)	0.0343 (11)	0.0297 (10)	−0.0051 (8)	−0.0011 (8)	−0.0014 (8)
C6	0.0254 (9)	0.0279 (10)	0.0253 (10)	0.0013 (8)	0.0032 (7)	0.0019 (8)
C7	0.0279 (9)	0.0261 (10)	0.0230 (9)	−0.0014 (7)	0.0046 (7)	−0.0037 (7)
C8	0.0345 (10)	0.0259 (10)	0.0311 (10)	0.0018 (8)	0.0047 (8)	−0.0016 (8)
C9	0.0429 (12)	0.0298 (10)	0.0326 (11)	−0.0095 (9)	0.0079 (9)	−0.0016 (8)
C10	0.0308 (10)	0.0416 (12)	0.0330 (11)	−0.0120 (9)	0.0038 (8)	−0.0045 (9)
C11	0.0284 (10)	0.0391 (11)	0.0305 (11)	−0.0017 (8)	−0.0007 (8)	−0.0016 (9)
C12	0.0295 (10)	0.0289 (10)	0.0269 (10)	−0.0012 (8)	0.0020 (8)	−0.0008 (8)
C13	0.0236 (9)	0.0238 (9)	0.0234 (9)	0.0015 (7)	0.0022 (7)	−0.0014 (7)
C14	0.0233 (9)	0.0221 (9)	0.0278 (10)	−0.0008 (7)	−0.0011 (7)	0.0005 (7)
C15	0.0272 (9)	0.0294 (10)	0.0297 (10)	0.0020 (8)	0.0012 (8)	0.0044 (8)
C16	0.0359 (11)	0.0272 (10)	0.0302 (11)	0.0034 (8)	0.0020 (8)	0.0050 (8)
C17	0.0373 (11)	0.0256 (10)	0.0333 (11)	−0.0042 (8)	0.0060 (8)	0.0005 (8)
C18	0.0245 (9)	0.0306 (10)	0.0387 (11)	−0.0020 (8)	0.0021 (8)	−0.0066 (9)
C19	0.0260 (9)	0.0254 (9)	0.0310 (10)	0.0030 (7)	−0.0009 (8)	−0.0012 (8)
C20	0.0273 (9)	0.0311 (10)	0.0276 (10)	0.0019 (8)	0.0030 (8)	0.0009 (8)
C21	0.0282 (10)	0.0274 (10)	0.0358 (11)	0.0021 (8)	0.0001 (8)	0.0071 (8)
C22	0.0270 (9)	0.0217 (9)	0.0397 (11)	0.0019 (7)	0.0033 (8)	0.0004 (8)
C23	0.0390 (11)	0.0298 (11)	0.0316 (11)	0.0032 (8)	0.0034 (8)	−0.0024 (8)
C24	0.0375 (11)	0.0263 (10)	0.0251 (10)	0.0025 (8)	0.0007 (8)	0.0026 (8)

### trans-3-Benzoyl-2,2-diphenyl-4-(trifluoromethyl)tetrahydrothiophene (1b) Geometric parameters (Å, º)

**Table:**

S1—C2	1.8567 (18)	C11—C12	1.387 (3)
S1—C5	1.799 (2)	C11—H11	0.9500
F1—C1	1.349 (2)	C12—H12	0.9500
F2—C1	1.341 (2)	C13—C15	1.394 (3)
F3—C1	1.343 (2)	C13—C19	1.399 (2)
O1—C6	1.215 (2)	C14—C20	1.388 (3)
C1—C4	1.503 (3)	C14—C24	1.401 (3)
C2—C3	1.590 (2)	C15—C16	1.386 (3)
C2—C13	1.534 (2)	C15—H15	0.9500
C2—C14	1.537 (2)	C16—C17	1.380 (3)
C3—C4	1.544 (2)	C16—H16	0.9500
C3—C6	1.532 (3)	C17—C18	1.388 (3)
C3—H3	1.0000	C17—H17	0.9500
C4—C5	1.533 (3)	C18—C19	1.381 (3)
C4—H4	1.0000	C18—H18	0.9500
C5—H51	0.9900	C19—H19	0.9500
C5—H52	0.9900	C20—C21	1.394 (3)
C6—C7	1.495 (3)	C20—H20	0.9500
C7—C12	1.393 (3)	C21—C22	1.378 (3)
C7—C8	1.399 (3)	C21—H21	0.9500
C8—C9	1.391 (3)	C22—C23	1.389 (3)
C8—H8	0.9500	C22—H22	0.9500
C9—C10	1.384 (3)	C23—C24	1.381 (3)
C9—H9	0.9500	C23—H23	0.9500
C10—C11	1.385 (3)	C24—H24	0.9500
C10—H10	0.9500		
			
C2—S1—C5	90.00 (8)	C9—C10—H10	119.8
F2—C1—F3	106.23 (15)	C11—C10—H10	119.8
F2—C1—F1	106.26 (16)	C10—C11—C12	119.89 (19)
F3—C1—F1	105.88 (16)	C10—C11—H11	120.1
F2—C1—C4	112.36 (16)	C12—C11—H11	120.1
F3—C1—C4	111.89 (16)	C11—C12—C7	120.45 (18)
F1—C1—C4	113.67 (16)	C11—C12—H12	119.8
S1—C2—C3	103.16 (11)	C7—C12—H12	119.8
S1—C2—C13	109.24 (12)	C15—C13—C19	117.66 (17)
S1—C2—C14	107.10 (12)	C15—C13—C2	122.06 (16)
C3—C2—C13	113.37 (14)	C19—C13—C2	120.22 (16)
C3—C2—C14	113.04 (15)	C20—C14—C24	117.52 (17)
C13—C2—C14	110.44 (14)	C20—C14—C2	125.20 (17)
C6—C3—C4	111.45 (15)	C24—C14—C2	117.28 (16)
C6—C3—C2	112.12 (14)	C16—C15—C13	120.87 (18)
C2—C3—C4	108.88 (14)	C16—C15—H15	119.6
C6—C3—H3	108.1	C13—C15—H15	119.6
C4—C3—H3	108.1	C17—C16—C15	120.70 (18)
C2—C3—H3	108.1	C17—C16—H16	119.7
C1—C4—C5	112.39 (16)	C15—C16—H16	119.7
C1—C4—C3	112.68 (15)	C16—C17—C18	119.30 (18)
C3—C4—C5	109.35 (15)	C16—C17—H17	120.4
C1—C4—H4	107.4	C18—C17—H17	120.4
C5—C4—H4	107.4	C19—C18—C17	120.09 (18)
C3—C4—H4	107.4	C19—C18—H18	120.0
S1—C5—C4	102.77 (13)	C17—C18—H18	120.0
C4—C5—H51	111.2	C18—C19—C13	121.38 (18)
S1—C5—H51	111.2	C18—C19—H19	119.3
C4—C5—H52	111.2	C13—C19—H19	119.3
S1—C5—H52	111.2	C14—C20—C21	120.96 (18)
H51—C5—H52	109.1	C14—C20—H20	119.5
O1—C6—C7	121.13 (17)	C21—C20—H20	119.5
O1—C6—C3	120.47 (16)	C22—C21—C20	121.03 (18)
C7—C6—C3	118.37 (15)	C22—C21—H21	119.5
C12—C7—C8	119.17 (17)	C20—C21—H21	119.5
C12—C7—C6	123.27 (17)	C21—C22—C23	118.44 (18)
C8—C7—C6	117.56 (16)	C21—C22—H22	120.8
C9—C8—C7	120.16 (18)	C23—C22—H22	120.8
C9—C8—H8	119.9	C24—C23—C22	120.84 (19)
C7—C8—H8	119.9	C24—C23—H23	119.6
C10—C9—C8	119.86 (19)	C22—C23—H23	119.6
C10—C9—H9	120.1	C23—C24—C14	121.20 (18)
C8—C9—H9	120.1	C23—C24—H24	119.4
C9—C10—C11	120.43 (18)	C14—C24—H24	119.4
			
C5—S1—C2—C13	−161.09 (13)	C8—C9—C10—C11	0.2 (3)
C5—S1—C2—C14	79.28 (13)	C9—C10—C11—C12	1.4 (3)
C5—S1—C2—C3	−40.22 (12)	C10—C11—C12—C7	−1.0 (3)
C13—C2—C3—C6	15.9 (2)	C8—C7—C12—C11	−0.9 (3)
C14—C2—C3—C6	142.53 (15)	C6—C7—C12—C11	178.02 (17)
S1—C2—C3—C6	−102.15 (14)	C14—C2—C13—C15	150.47 (17)
C13—C2—C3—C4	139.65 (15)	C3—C2—C13—C15	−81.5 (2)
C14—C2—C3—C4	−93.67 (17)	S1—C2—C13—C15	32.9 (2)
S1—C2—C3—C4	21.64 (16)	C14—C2—C13—C19	−32.4 (2)
F2—C1—C4—C5	−59.3 (2)	C3—C2—C13—C19	95.64 (19)
F3—C1—C4—C5	−178.69 (15)	S1—C2—C13—C19	−149.93 (15)
F1—C1—C4—C5	61.4 (2)	C13—C2—C14—C20	118.39 (19)
F2—C1—C4—C3	176.63 (15)	C3—C2—C14—C20	−9.8 (3)
F3—C1—C4—C3	57.2 (2)	S1—C2—C14—C20	−122.75 (17)
F1—C1—C4—C3	−62.7 (2)	C13—C2—C14—C24	−60.3 (2)
C6—C3—C4—C1	−98.19 (18)	C3—C2—C14—C24	171.47 (15)
C2—C3—C4—C1	137.62 (16)	S1—C2—C14—C24	58.53 (19)
C6—C3—C4—C5	136.06 (16)	C19—C13—C15—C16	0.4 (3)
C2—C3—C4—C5	11.9 (2)	C2—C13—C15—C16	177.58 (17)
C1—C4—C5—S1	−167.14 (13)	C13—C15—C16—C17	0.0 (3)
C3—C4—C5—S1	−41.22 (17)	C15—C16—C17—C18	−0.6 (3)
C2—S1—C5—C4	47.49 (13)	C16—C17—C18—C19	0.9 (3)
C4—C3—C6—O1	−23.0 (2)	C17—C18—C19—C13	−0.5 (3)
C2—C3—C6—O1	99.3 (2)	C15—C13—C19—C18	−0.1 (3)
C4—C3—C6—C7	158.71 (16)	C2—C13—C19—C18	−177.36 (17)
C2—C3—C6—C7	−78.9 (2)	C24—C14—C20—C21	−0.7 (3)
O1—C6—C7—C12	151.51 (19)	C2—C14—C20—C21	−179.45 (17)
C3—C6—C7—C12	−30.3 (3)	C14—C20—C21—C22	0.1 (3)
O1—C6—C7—C8	−29.5 (3)	C20—C21—C22—C23	0.5 (3)
C3—C6—C7—C8	148.70 (17)	C21—C22—C23—C24	−0.5 (3)
C12—C7—C8—C9	2.5 (3)	C22—C23—C24—C14	−0.2 (3)
C6—C7—C8—C9	−176.52 (17)	C20—C14—C24—C23	0.8 (3)
C7—C8—C9—C10	−2.1 (3)	C2—C14—C24—C23	179.60 (18)
